# Ischemic Lesion Growth in Patients with a Persistent Target Mismatch After Large Vessel Occlusion

**DOI:** 10.1007/s00062-022-01180-z

**Published:** 2022-07-05

**Authors:** Shinya Tomari, Thomas Lillicrap, Carlos Garcia-Esperon, Yumi Tomari Kashida, Andrew Bivard, Longting Lin, Christopher R. Levi, Neil J. Spratt

**Affiliations:** 1 Hunter Medical Research Institute, University of Newcastle, Newcastle, Australia; 2 Department of Neurology, John Hunter Hospital, Newcastle, Australia; 3 College of Health, Medicine, and Wellbeing, University of Newcastle, Newcastle, Australia; 4 Melbourne Brain Center at the Royal Melbourne Hospital, University of Melbourne, Melbourne, Australia

**Keywords:** Persistent large penumbra, Little core growth, Good collateral flow, CT perfusion image, CT perfusion collateral index

## Abstract

**Background:**

Failure to reperfuse a cerebral occlusion resulting in a persistent penumbral pattern has not been fully described.

**Methods:**

We retrospectively reviewed patients with anterior large vessel occlusion who did not receive reperfusion, and underwent repeated perfusion imaging, with baseline imaging < 6 h after onset and follow-up scans from 16–168 h. A persistent target mismatch (PTM) was defined as core volume of < 100 mL, mismatch ratio > 1.2, and mismatch volume > 10 mL on follow-up imaging. Patients were divided into PTM or non-PTM groups. Ischemic core and penumbral volumes were compared between baseline and follow-up imaging between the two groups, and collateral flow status assessed using CT perfusion collateral index.

**Results:**

A total of 25 patients (14 PTM and 11 non-PTM) were enrolled in the study. Median core volumes increased slightly in the PTM group, from 22 to 36 ml. There was a much greater increase in the non-PTM group, from 57 to 190 ml. Penumbral volumes were stable in the PTM group from a median of 79 ml at baseline to 88 ml at follow-up, whereas penumbra was reduced in the non-PTM group, from 120 to 0 ml. Collateral flow status was also better in the PTM group and the median collateral index was 33% compared with 44% in the non-PTM group (*p* = 0.043).

**Conclusion:**

Multiple patients were identified with limited core growth and large penumbra (persistent target mismatch) > 16 h after stroke onset, likely due to more favorable collateral flow.

**Supplementary Information:**

The online version of this article (10.1007/s00062-022-01180-z) contains supplementary material, which is available to authorized users.

## Introduction

A total of five randomized controlled trials revealed the safety and efficacy of endovascular thrombectomy (EVT) for acute ischemic stroke with large vessel occlusion in the anterior circulation within 6–12 h after symptom onset [1–5]. Core growth pattern after large vessel occlusion is divided into fast or slow progress [6]. Fast progressors have worse outcomes when receiving EVT beyond 6 h of onset than those who received EVT within 6 h [7]. The DAWN and DEFUSE3 trials demonstrated the efficacy of EVT in the 16–24 h window, in patients with a persistent favorable clinical or imaging mismatch pattern [8, 9]. Enrolled patients showed small infarct core volume and a large persistent target mismatch (PTM), representing potentially salvageable ischemic penumbra, identified either using perfusion imaging [9], or from brain functional impairment, represented as a significant neurological deficit on The National Institute of Health Stroke Scale (NIHSS) [8]. These findings provide very strong evidence of persistent salvageable ischemic brain tissue after many hours. Interestingly, there was little influence of time to treatment on outcome in these studies [8, 9], suggesting that in some patients salvageable penumbra and benefit from EVT are likely to persist beyond 24 h. Previous studies showed that good collateral flow was correlated with small infarct volume, a PTM pattern, and better clinical outcomes [10, 11]. However, there are very little data to indicate how fast ischemic core volume grows and how long significant volume of penumbra may remain after large vessel occlusion in patients with PTM.

The DAWN and DEFUSE 3 trials reported that patients treated with medicinal therapy suffered from poorer neurological outcomes than those treated with EVT. The median time from stroke onset to imaging in those trials was 10–13 h, thus whether significant at-risk penumbra may persist beyond 24 h, and for how long, remains largely unknown. However, in both trials, there was no suggestion of declining efficacy of EVT with increasing time, supporting that in some patients at least, it does persist for up to 16–24 h, and maybe longer [8, 9, 12]. The aims of this study were a) to assess core volume growth and persistent penumbra volume after large vessel occlusion in PTM patients, and b) to investigate neurological outcomes in PTM patients.

## Methods

### Neuroimaging

Computed tomography (CT) imaging was derived from 320-slice Aquilion ONE scanner (Canon medical systems, Otawara, Japan). Image scanning started 7 s after intravenous injection (40 ml, injected at 6 ml/s) of non-ionic iodinated contrast (Ultravist 370; Bayer HealthCare, Berlin, Germany). It lasted for 60 s, acquiring 19 images per slice. Magnetic resonance perfusion (MRP) image was performed on a 1.5-Tesla scanner (Siemens Avanto, Erlangen, Germany) and included axial isotropic diffusion weighted image (DWI), echoplanar spin-echo sequence, time of flight magnetic resonance angiography, and bolus-tracking perfusion-weighted imaging. Following a bolus of gadolinium contrast (Magnevist; Bayer HealthCare) into the antecubital vein (0.2 mmol/kg, injected at speed of 5 ml/s), perfusion images were obtained with acquisition parameters. The scanning lasted for 60 s, resulting in 40 images per slice. A total 19 slices were obtained. CT perfusion (CTP) and MRP imaging were post-processed using the commercial software MIStar (Apollo Medical Imaging Technology, Melbourne, Australia) to generate automated core-penumbra maps. Penumbra was defined as the tissue with a delay time (DT) ≥ 3 s and relative cerebral blood flow (CBF) ≥ 30% of the contralateral hemisphere using either CTP or MRP in MISTar [13]. Ischemic core was defined as the tissue with a DT ≥ 3 s and a relative CBF ≤ 30% of the contralateral hemisphere or an apparent diffusion coefficient threshold of < 620 × 10^−6^ mm^2^/s on MRI-DWI. All images were reprocessed and analyzed using the same version of the MIStar software (Apollo Medical Imaging Technology, Melbourne, Australia, Version 3.2 release 3.2.62.03, last update October 2019).

### Population and Data Collection

We retrospectively enrolled acute ischemic stroke patients presenting to the John Hunter Hospital (New South Wales, Australia) from April 2011 to April 2017 who underwent acute CTP imaging at baseline (within 6 h after stroke onset or 12 h after last known well [LSW]), with follow-up CTP or MRP imaging performed between 16 and 168 h after onset. We included patients who had large vessel occlusion (internal carotid artery [ICA], middle cerebral artery [MCA], segment 1) without effective reperfusion. The on-call radiologist (consultant or resident) assessed CT angiography initially and a senior radiologist confirmed the report in the clinical practice. S.T. (stroke neurologist, > 10 years of experience) read the CT angiography with the report and the perfusion image to assess the reperfusion in this study. Effective reperfusion was defined as a > 80% reduction in the volume of the perfusion lesion (DT threshold > 3 s [13]) from baseline to 24 h imaging [14]. Patients with a ratio of perfusion lesion to infarct core volume < 1.2 at baseline imaging were excluded. PTM was defined as an ischemic core volume of < 100 mL, a ratio of perfusion lesion to infarct core volume > 1.2, and a mismatch volume between core volume and perfusion lesion > 10 mL at follow-up imaging, by modifying the inclusion criteria of EXTEND-IA trial [2]. The study had ethical institutional approval, and written informed consent was obtained from each patient as part of INSPIRE research.

### Assessment

Patients were divided into PTM or non-PTM groups. We measured core and penumbra volumes at baseline and follow-up imaging for each group, and calculated core volume growth (core volume at follow-up—core volume at baseline). We compared image findings (core and penumbra volumes, time from stroke onset or LSW to image, occlusion site, hemorrhagic transformation), clinical characteristics (age, sex, premorbid modified Rankin Scale [mRS], risk factors, premedication, stroke etiology, blood pressure, thrombolysis, NIHSS scores at baseline and follow-up [assessed at 24–72 h after stroke onset]), and neurological outcomes (mRS at 3 months after stroke onset; 0–2 as favorable).

### CTP Collateral Index

We compared CTP collateral index (CTPCI) between the two groups at baseline imaging. The CTPCI is a continuous variable with lower value representing better collateral flow [15]. The following equation was used to define the index: CTPCI = DT > 6 / DT > 2.

**Fig. 1:**
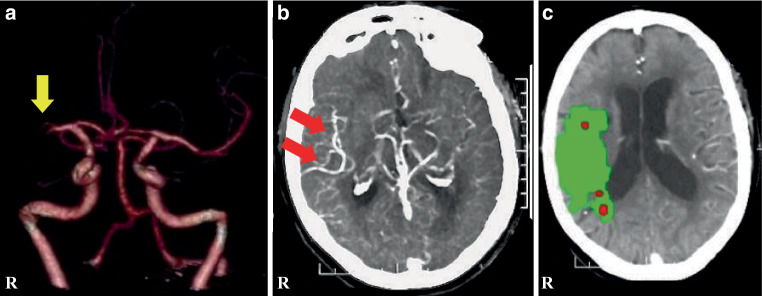
Representative case presentation; a patient had right middle cerebral artery proximal occlusion. Each image was performed at 2 h after stroke onset. **a** 3D-CT angiography; *yellow arrow* shows right middle cerebral artery proximal occlusion. **b** Dynamic CT angiography; *red arrows* show vessels reconstituted by collateral flow. **c** CT perfusion image; *green area* shows hypoperfusion lesion (90 ml) and *red area* shows ischemic core lesion (6 ml). Mismatch volume (penumbra lesion) was 84 ml. CT perfusion collateral index: (delay time > 6 s lesion 21 ml/delay time > 2 s lesion 124 ml) × 100 = 17

DT > 6 s measures the severe hypoperfusion region without collateral flow, whereas DT > 2 s defines the region with any hypoperfusion on CTP. The index has a significant correlation with dynamic CT angiography collateral scores with an optimal cut point of 31.8% in predicting good collateral status ([15]; Fig. 1).

### Statistical Analysis

Continuous variables were presented as median with interquartile range. Clinical characteristics were compared between PTM and non-PTM patients using Pearson’s χ^2^-test for categorical variables, Student’s unpaired *t-*test was used for normally distributed continuous variables and Wilcoxon’s rank sum test for non-normally distributed continuous variables. Variables with a *P*-value < 0.05 in the univariate analysis were selected for the logistic regression model to adjust the main analysis for thrombolysis. All statistical analysis was done on STATA 15.0 (Stata Corp, College Station, TX, USA) with significance level set at 0.05.

### Data Availability

The datasets generated for this study are available on reasonable request to the corresponding author.

## Results

We reviewed 237 patients who had baseline and follow-up perfusion images within 1 week after stroke onset. We excluded those with small vessel occlusion (*n* = 63), posterior fossa stroke (*n* = 9), reperfusion (*n* = 98), critical data not available (*n* = 29), and follow-up image performed within 16 h (*n* = 13) cases. We enrolled 25 patients (21 were from between 2011–2014 and 4 between 2015–2017) with follow-up perfusion scan beyond 16 h after onset. All patients underwent CTP at baseline, 7 had CTP and 18 MRP at follow-up imaging. There were no patients who had a history of intracranial atherosclerotic disease in the study cohort as determined during the patient’s in-hospital stay and no patients with the concomitant intracranial atherosclerotic disease found on the CT angiography.

Fourteen patients were classified as the PTM group, while 11 patients as the non-PTM group. Stroke onset time was unknown in six patients (two PTM and four non-PTM) but the time of LSW were all available. Just one non-PTM patient underwent EVT, unsuccessfully. Table 1 shows a comparison of clinical characteristics and image findings between the two groups. PTM patients were a median of 67 years old, compared with a median of 69 years in non-PTM patients. More than 90% in both groups were independent (mRS 0–1) before stroke. There were no significant differences in risk factors or stroke etiology between the two groups. Median time from onset to baseline imaging was 2 h in both groups, and the time to follow-up imaging in the PTM group was 24 h (range 18–78 h), versus 28 h (range 19–54 h) in the non-PTM group. The PTM group had 3 ICA occlusion (2 terminus and 1 tandem) and 11 M1 occlusion, while the non-PTM group had 8 ICA occlusion (7 terminus and 1 tandem) and 3 M1 occlusion. In the PTM group, the median CTP collateral index was 33%, compared with 44% in the non-PTM group (*p* = 0.043). The logistic regression model showed that thrombolysis had an odds ratio of 1.6 to the PTM group with a *P*-value of 0.70 and a 95% confidence interval of 0.15–16.52 (Supplementary material).

**Table 1:** Comparison of clinical characteristics and image findings between the PTM and non-PTM groups

	PTM *n* = 14	Non-PTM *n* = 11	*p*-value
**Clinical findings**			
*Age, years, median [IQR]*	67 [55–72]	69 [63–73]	0.55
*Sex, men, no. (%)*	9 (64)	8 (73)	0.65
*Premorbid mRS 0–1, no. (%)*	13 (93)	10 (91)	0.92
*Risk factors, no. (%)*			
Hypertension	4 (29)	7 (64)	0.08
Dyslipidemia	4 (29)	3 (27)	0.94
Diabetes mellitus	1 (7.1)	1 (9.1)	0.86
Atrial fibrillation	7 (50)	3 (27)	0.25
Current smoking	3 (21)	2 (18)	0.84
*Pre-antiplatelet or anticoagulant therapy, no. (%)*	3 (21)	1 (9.1)	0.40
*Stroke etiology, no. (%)*			
Cardioembolism	9 (64)	5 (45)	0.35
Atherosclerosis	1 (7.1)	4 (36)	0.07
Unknown	4 (29)	2 (18)	0.55
*Blood pressure, mm* *Hg, median [IQR]*			
Systolic	140 [134–150]	160 [140–182]	0.13
Diastolic	78 [65–90]	90 [80–98]	0.25
*Baseline NIHSS, median [IQR]*	15 [10–19]	18 [17–22]	0.10
*Follow-up NIHSS, median [IQR]*	15 [10–18]	19 [18–21]	0.018
*Thrombolysis, no. (%)*	11 (79)	6 (55)	0.20
**Image findings**			
*Time from onset or LSW to image, hours, median [IQR]*			
To baseline	2.1 [1.3–2.8]	2.0 [1.6–2.7]	0.81
To follow-up	24 [20–30]	28 [25–39]	0.25
*Occlusion site, no. (%)*			
ICA	3 (21)	8 (73)	0.01
MCA (M1 segment)	11 (79)	3 (27)	–
*Hemorrhagic transformation, no. (%)*	0	2 (18)	0.096
*CTP collateral index, median [IQR]*	33 [18–36]	44 [29–47]	0.043

*PTM* persistent target mismatch, *IQR* interquartile range, *mRS* modified Rankin Scale, *NIHSS* National Institutes of Health stroke scale, *LSW* last seen well, *ICA* internal carotid artery, *MCA* middle cerebral artery, *CTP* computed tomography perfusion

**Fig. 2:**
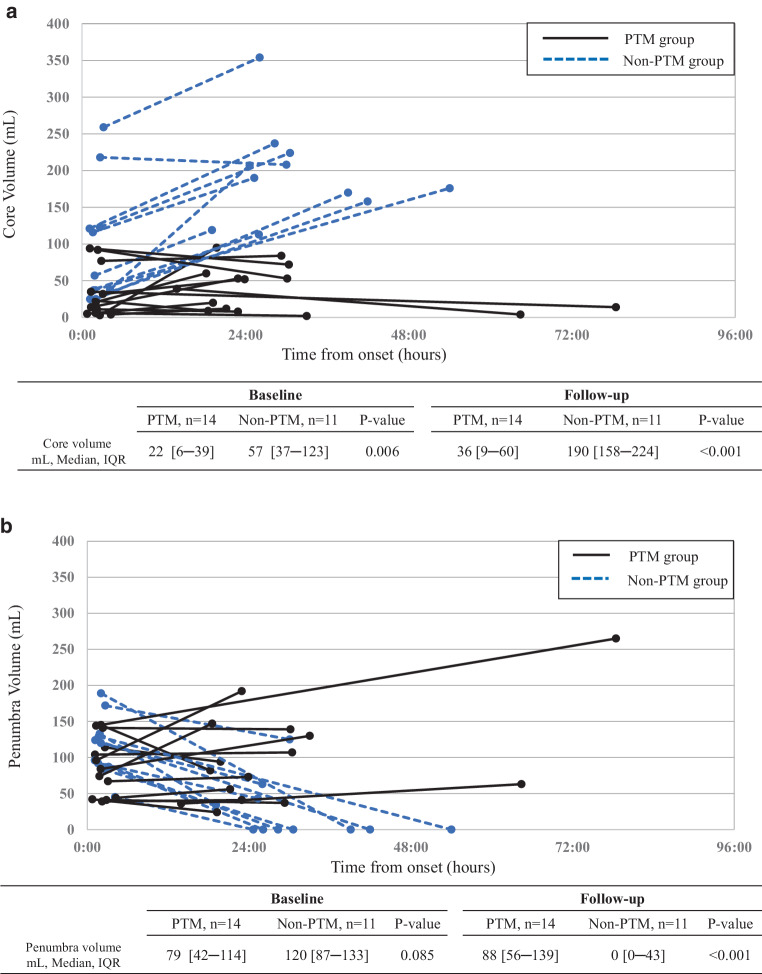
**a** Core growth between baseline and follow-up perfusion images in the PTM and the non-PTM groups. **b** Penumbra volume change between baseline and follow-up perfusion images in the persistent target mismatch (PTM) and the non-PTM groups. *IQR* Interquartile range

**Fig. 3:**
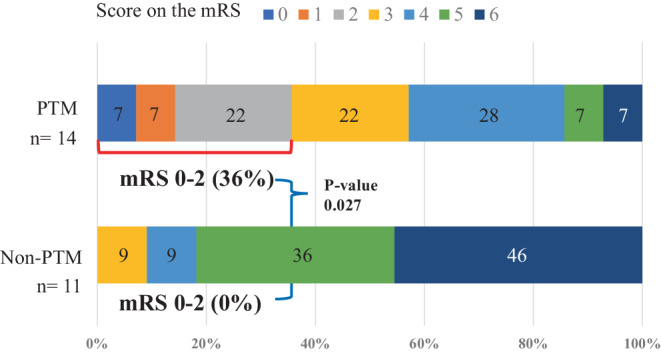
Modified Rankin Scale (mRS) at 3 months after stroke between the PTM and the non-PTM groups. *PTM* persistent target mismatch

Figure 2a shows core growth between baseline and follow-up imaging in the two groups. The PTM group had smaller core volume than the non-PTM group at baseline imaging (the PTM group median 22 ml, versus the non-PTM group median 57 ml, *p* = 0.006), and also at follow-up imaging (median 36 ml versus median 190 ml, *p* < 0.001). PTM patients had minimal core volume growth between imaging, while all but one patient in the non-PTM group had core volume growth (median 2 ml versus 101 ml, *P* < 0.001). Figure 2b shows penumbra volume between baseline and follow-up imaging in the two groups. The PTM group had smaller penumbra volume than the non-PTM group at baseline (median 79 ml versus median 120 ml, *p* = 0.085). At follow-up imaging, the PTM patients retained large penumbra volumes, including five increased cases, while all non-PTM patients had decreased penumbra volume (median 88 ml versus median 0 ml, *p* < 0.001). Figure 3 shows the mRS at 3 months after stroke between the two groups. The PTM group had better clinical outcome than the non-PTM group (median 3 of mRS versus median 5, *p* = 0.015 and 36% of proportion of mRS 0–2 versus 0%, *p* = 0.027). More than 80% of the non-PTM patients suffered from severe neurological outcomes, with mRS 5–6.

## Discussion

We identified 14 patients with a PTM pattern after large vessel occlusion, in whom follow-up perfusion imaging was performed a median of 24 h later. The PTM group demonstrated the small growth of the infarct core and the persistent large penumbra at the time of repeat imaging, and had the better clinical outcome.

M1 occlusion was more frequent in the PTM group, while ICA was the major occlusion site in the non-PTM group (all but one were ICA terminus). This suggests that core growth with M1 occlusion was slower than that with ICA occlusion, which was consistent with the results in previous studies [7, 16]. Willisian collateral failure (blockage of flows from anterior and posterior communicating arteries) due to ICA terminus occlusion [17] might explain whole poorer collateral flow compared with M1 occlusion, which can benefit from Willisian collaterals.

Good collateral flow has been associated with reduced core growth [18–20] and good clinical outcome [21]. We have reported that CTP can accurately quantify collateral flow after acute ischemic stroke [15], and that collateral status is a major determinant for core growth rate (low CTP collateral index implies good collateral flow) [22]. PTM patients had a median collateral index of 33%, significantly better than that seen in the non-PTM group. Better collateral flow has been related to lower infarct growth rate [23, 24] and larger persistent penumbra volume in untreated patients within 24 h of stroke onset [25]. We found a similar result, despite half of the included patients having received follow-up imaging ≥ 24 h from symptom onset.

An important implication of this study is that clinicians may be able to predict from the CTPCI on perfusion imaging performed in the first 6 h, which patients are very likely to have persistent penumbra beyond 16 h. Such patients are likely to benefit from transfer to a comprehensive stroke centre for thrombectomy, even over very long distances if necessary and possible. In keeping with the lack of effect of time to treatment in DEFUSE 3 and DAWN, subanalyses revealed no significant difference in clinical outcomes between patients with direct admission for thrombectomy and transferred patients [26, 27].

In our study, the PTM group had the higher proportion of mRS 0–2 at 3 months than the non-PTM group. The PTM patients also had better collateral status that likely contributed to the more favourable outcome. This suggests that the presence of a PTM pattern on imaging even 18–78 h after symptom onset, is predictive of a better outcome, even in the absence of treatment.

Nonetheless, the PTM group did not have as good outcomes as EVT groups in DEFUSE 3 (mRS 0–2 at 3 months: 45%) and in DAWN (52%) trials [8, 9]. The time from onset to follow-up imaging in the PTM group (median 24 h) was longer than in those trials (median 10–13 h) and it remains to be established whether reperfusion will improve outcomes in such patients. However, previous case control studies reported patients with a PTM pattern had good outcomes after thrombectomy performed even beyond 24 h (mRS 0–2 at 3 months; 43% and 54%)[28, 29]. These studies boost the trend of tissue-based assessment rather than time-based one, but clinical trials are needed to test the efficacy of thrombectomy beyond 24 h.

Strengths of this study are that we assessed ischemic core and penumbra volume with baseline and follow-up perfusion imaging, and all perfusion images were analyzed with the same software (MISTar). Of 14 PTM patients, 12 had clear stroke onset time. There are several limitations. The first one is the small sample size because of the retrospectivity. Secondly, just 1 patient underwent EVT since 21 patients were recruited before 2015 when the efficacy of EVT was established, and only a single neurointerventionalist was available at our hospital during the study period. Finally, we did not distinguish penumbra and benign oligemia. However, CT perfusion distinguishes benign oligemia and true ischemic penumbra [30] and the thresholds used by the research team are well validated [31] and have been reproduced multiple times by external groups [32, 33], and have been demonstrated not to overestimate the perfusion lesion volume.

Clinical implications of this study are that patients with good collateral flow may have a persistent penumbra lesion which suggests the chance of reperfusion with EVT in a very late time window. Patients with low (good) CTP collateral index at primary centers may have a chance to receive EVT at comprehensive stroke centers after long transfer, even beyond 24 h after stroke onset.

## Conclusion

The PTM patients had slow core growth and large volumes of persistent penumbra even 18–78 h after stroke onset, associated with good collateral flow. Such patients had significantly better 3‑month neurological outcomes than those without a PTM pattern, but still not as good as those seen in the treatment arms of the late-window EVT trials. Such patients would be good candidates for trials of EVT in a very late time window, beyond 16–24 h.

## Supplementary Information

Table I. Logistic regression model of predictors of the persistent target mismatch
