# Supplementary material for: Ischemic Lesion Growth in Patients with a Persistent Target Mismatch After Large Vessel Occlusion
Source: Clin Neuroradiol. 2022 Jul 5;33(1):41–8. doi: 10.1007/s00062-022-01180-z (PMC10014761; doi:10.1007/s00062-022-01180-z)
Supplement: Supplementary file 1 — Table I. Logistic regression model of predictors of the persistent target mismatch [file 62_2022_1180_MOESM1_ESM.docx]

**Supplementary material**

Table I. Logistic regression model of predictors of the persistent target mismatch

|  | Odds ratio | P-value | 95% confidence interval |
| --- | --- | --- | --- |
| Thrombolysis | 1.6 | 0.70 | 0.15─16.53 |
| ICA occlusion | 0.09 | 0.035 | 0.009─0.84 |
| CTP collateral index, % | 0.9* | 0.065 | 0.82─1.01 |

ICA; internal carotid artery, CTP; computed tomography perfusion

*for each one percent increase in CTP collateral index
